# Assessment of physical activity levels and back pain among poles and Portuguese in the further year of the COVID-19 pandemic - a pilot study

**DOI:** 10.1186/s12889-024-18088-7

**Published:** 2024-02-23

**Authors:** Monika Gałczyk, Anna Zalewska, Marek Sobolewski, Hélder Fernandes

**Affiliations:** 1 Faculty of Health Sciences, University of Lomza, 14 Akademicka St., 18-400 Lomza, Poland; 2 Plant of Quantitative Methods, Rzeszow University of Technology, al. Powstancow Warszawy 12, 35-959 Rzeszow, Poland; 3 Health Sciences Research Unit: Nursing (UICISA: E), Instituto Politécnico de Bragança, 5300-253 Bragança, Portugal

**Keywords:** Neck pain, Low back pain, Physical exertion, COVID-19, Poland, Portugal

## Abstract

**Background:**

The vast majority of people have experienced the problem of back pain at least once in their lives for a variety of reasons. It is well known that regular physical activity at a moderate level has a number of health benefits regardless of age or gender with positive effects on the musculoskeletal, cardiovascular, respiratory or nervous systems improving fitness levels. During the pandemic, Poland experienced one of the longest periods of school lockdown in Europe. In Portugal, this period was considerably shorter. The aim of this study was to determine the level of physical activity and back pain as well as relationship between physical activity and back pain in a group of Polish and Portuguese students in the following year the COVID-19 pandemic.

**Methods:**

The study was conducted via the Internet among students from Poland and Portugal (398 respondents − 229 from Poland and 169 from Portugal) in October 2022. In the pilot study, the International Physical Activity Questionnaire and The Oswestry Disability Index and Neck Disability Index questionnaires were used to assess the level of back pain.

**Results:**

There are no statistically significant differences in the level of physical activity and pain complaints of respondents from the two countries. At least half of the students do not report any pain complaints (median in some groups being 0). In the Portuguese men, there is a correlation between the level of physical activity and the Neck Disability Index score (*p* = 0.0304).

**Conclusions:**

The following year, after the pandemic COVID-19 was declared, the prevalence of back pain among students in Poland and Portugal is still significant.

## Introduction

The vast majority of people have experienced the problem of back pain at least once in their lives for a variety of reasons. In most cases, the cause of the occurrence of spinal problems is quite non-specific and related to economic and social, physical or psychological factors. Increasingly it is becoming more and more important public health problem, affecting the quality of life and at the same time being one of the reasons for work disability [1–4].

Another important reason related to the change in the lifestyle of the population in the last three years was the COVID-19 pandemic. Various safety measures were implemented worldwide to prevent the rapid spread of the disease. People were forced to wear masks, stay indoors, and limit direct contact with others [5–8]. All the restrictions imposed from above hindered people’s freedom of movement and physical activity, weakening the spine through various static-dynamic loads. These changes may have become another reason for the appearance of spinal pain mainly among people with chronic musculoskeletal problems [9].

It is well known that regular physical activity at a moderate level has a number of health benefits regardless of age or gender with positive effects on the musculoskeletal, cardiovascular, respiratory or nervous systems improving fitness levels [10, 11].

**Fig. 1:**
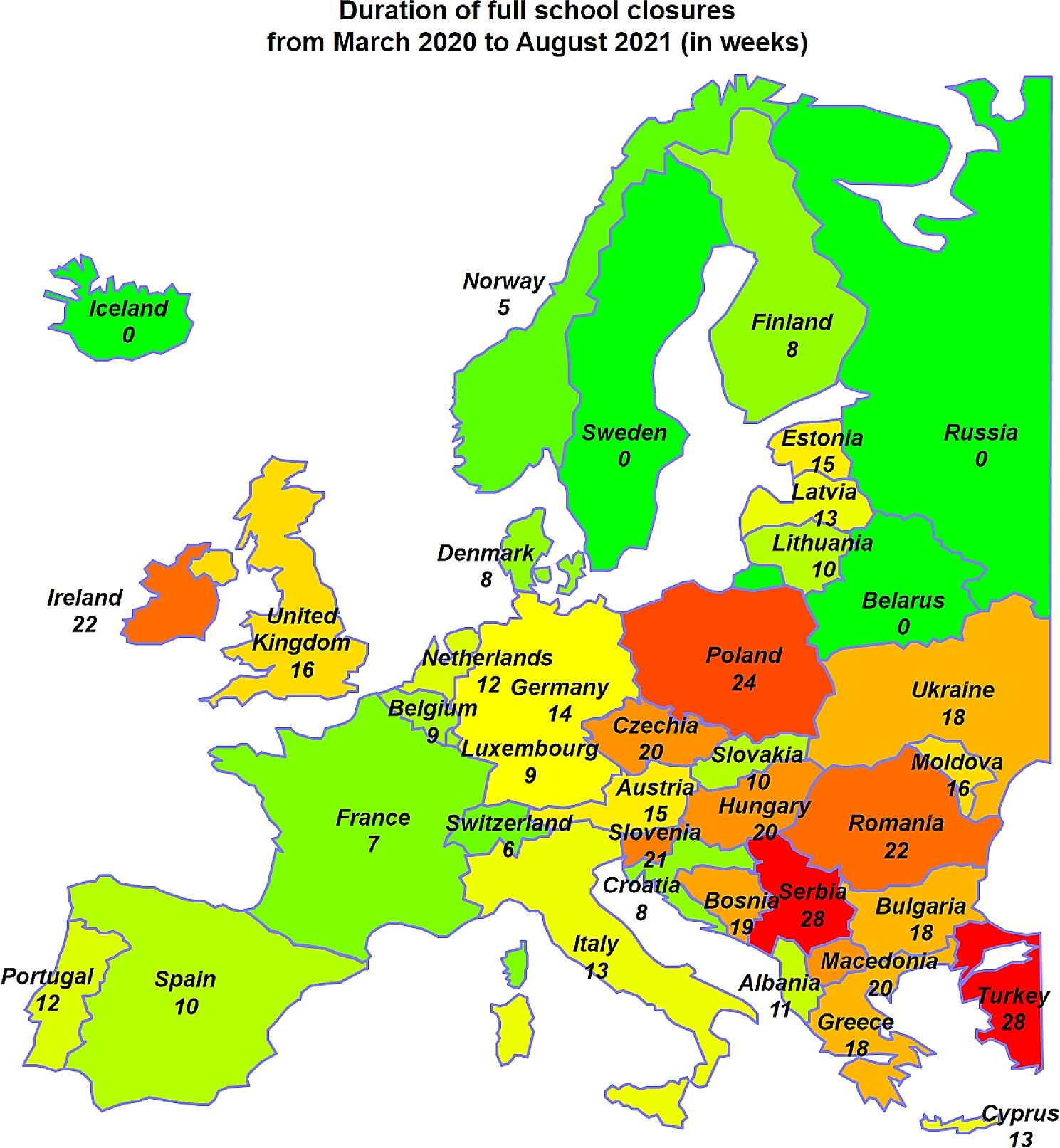
Duration of full school closures in chosen Europe countries from March 2020 to August 2021 (Countries with a long period of school closures are marked with shades of red, and those with the shortest duration of distance learning are marked with shades of green.) [19]

The relationship between reduced levels of physical activity associated with the imposed quarantine in most countries around the world and complaints of musculoskeletal pain is increasingly becoming a topic of increased interest among researchers. There are numerous studies, confirming the positive effects of physical activity through the use of mainly strengthening exercises to reduce spinal pain [12–15]. Students are reported to be most affected by reduced mobility as well as reduced levels of physical activity. Their limited daily activity is mainly due to the fact that they sit most of the day and often adopt an incorrect posture when performing their tasks. This often leads to musculoskeletal overload and the occurrence of cervical and lumbar spine pain [16–18]. The article is the result of international cooperation between authors from Poland and Portugal. During the pandemic, Poland experienced one of the longest periods of school lockdown in Europe. In Portugal, this period was considerably shorter (Fig. 1). Therefore, the authors decided to conduct a pilot study in this particular population. The aim of this study was to determine the level of physical activity and back pain as well as relationship between physical activity and back pain in a group of Polish and Portuguese students in the following year the COVID-19 pandemic.

## Materials and methods

### Participants and procedure

The survey was conducted among Polish and Portuguese students in October 2022. The authors provided links via the Internet to a Google form containing the questionnaire, with a request to complete it. The questionnaire was shared on social media by students from different fields of study. In addition, the questionnaire was accompanied by information about voluntary consent to the research and its anonymity. The questionnaire was prepared in Polish for the Polish student groups and in Portuguese for the Portuguese students. The criteria for inclusion in the study were: place of residence (either Poland or Portugal), consent to participate in the study, being currently enrolled, and complete completion of the questionnaire. On the other hand, the exclusion criteria were: no consent to participate in the study, incomplete completion of the questionnaire, residence in a country other than Poland or Portugal, non-student.

Because the key research question was to examine the relationship between activity (IPAQ) and pain (ODI and NDI), the sample size was determined to ensure adequate power in the significance test of the correlation coefficient (in each of the analyzed subgroups). Sample size for a Spearman correlation was determined using power analysis for: α = 0.05, 1-ß = 0.80, r_S_ = 0.30. Based on the this assumptions, the required sample size was 82. The three subgroups distinguished by country and gender were much larger, which guaranteed the rejection of the hypothesis of zero correlation with a power higher than 0.80. Only in the case of the group of men from Portugal, information was collected from only 51 people - in this case, the power of the significance test for the correlation coefficient was slightly lower and amounted to approximately 0.60.

### Methods for assessing physical activity and spinal pain

#### International physical activity questionnaire

To assess physical activity levels, the authors used a shortened version of the International Physical Activity Questionnaire (IPAQ), which is available in Polish and Portuguese. The questionnaire consists of 7 questions covering all types of physical activity in daily life. It is designed for people aged 15–69 years. The questionnaire covers activities that are performed for at least 10 min without a break. The types of activity are defined in the unit MET - min/week. The value for Cronbach’s alpha given in the publications is > 0.7 [20, 21].

#### Oswestry disability index and neck disability index

Spinal pain was assessed with an internationally used cervical spine disability questionnaire (NDI) and lumbar spine disability questionnaire (ODI). The test contains 10 questions about activities of daily living such as walking, sitting, standing, self-care and social life. Each answer is scored as follows:

- A − 0 points,
- B − 1 point,
- C − 2 points,
- D − 3 points,
- E − 4 points,
- F − 5 points.

The points are then added up; the maximum number of points is 50.

Disability rating scale:

- 0-4: none
- 5-14: slight
- 15-24: average
- 25-34: severe
- over 35: total

The value for Cronbach’s alpha reported in papers is > 0.7 [22, 23].

### Statistical methods

The statistical analysis was carried out using Statistica v. 13 (TIBCO Software Inc. (2017) Statistica (data analysis software system), version 13. The significance of differences in back pain severity (ODI, NDI) and physical activity level (IPAQ) between groups of Portuguese and Polish students was assessed using the Mann-Whitney test. The chi-square test of independence was used to identify variations in the percentage distribution of back pain severity and level of exercise. Levels of back discomfort and physical activity were correlated using Spearman’s rank correlation coefficient. The significance of discrepancies in the classification of activity level vs. level of back pain was determined using the Kruskal-Wallis test. The use of non-parametric techniques was required due to the non-normality of the distributions of the ODI, NDI, and IPAQ measures, all of which showed a very substantial right-handed asymmetry. For all statistical analyses, a significance level of 0.05 (*) was established; however, findings *p* < 0.01(**) and *p* < 0.001were also indicated.

## Results

### General characteristics

502 subjects completed the questionnaires, but 398 questionnaires were analysed. 104 questionnaires were incomplete or not completed in accordance with the instructions. 398 students from Poland and Portugal participated in the pilot study. Both populations were predominantly female, with a larger difference among Portuguese respondents (Table 1). The average age of Polish and Portuguese students surveyed was similar (median − 22 years).

**Table 1:** Sex of respondents

Sex	Country		Total
	Poland	Portugal	
woman	129 (56.3%)	118 (69.8%)	247
man	100 (43.7%)	51 (30.2%)	151
total	229	169	398

70% of Polish students suffered from COVID-19, as compared with almost 85% of Portuguese respondents (Table 2). The difference in the proportion of COVID-19 sufferers in both groups was statistically significant, on average, the number of COVID-19 sufferers in the Polish group was 1.10 and in the Portuguese group 1.36. Unfortunately, the authors have no way of assessing the reliability of these responses.

**Table 2:** COVID-19 disease

COVID-19 disease	Country (*p* = 0.0029**)	
	Poland (*N* = 229)	Portugal (*N* = 169)
0	65 (28.4%)	27 (16%)
1	85 (37.1%)	64 (37.9%)
2	69 (30.1%)	68 (40.2%)
3	10 (4.4%)	10 (5.9%)
Mean	1.1	1.36

*p* - test probability value calculated by the Mann-Whitney test, *p* < 0.01 (**)

### Comparison of pain complaints of students from both countries

The following two tables (Tables 3 and 4) provide an overview of the severity of back pain among respondents from both countries. There are no statistically significant differences between the respondents from the two countries in terms of pain severity. The median values should also be used when interpreting the ODI and NDI values, as the average values are inflated. With the exception of the NDI for Portugal, at least half of the students do not experience any pain.

**Table 3:** Complaints of low back pain

Country	ODI (*p* = 0.2594)						
	Mean	Median	Std. dev.	***c***_25_	***c***_75_	Min	Max
**Poland**	2.9	0	4.4	0	5	0	21
**Portugal**	2.9	0	4.8	0	5	0	21

*p* - test probability value calculated by the Mann-Whitney test; ODI - Oswestry Disability Index

**Table 4:** Complaints of neck pain

Country	NDI (*p* = 0.0757)						
	Mean	Median	Std. dev.	***c***_25_	***c***_75_	Min	Max
**Poland**	3.4	2	4.4	0	5	0	26
**Portugal**	3	0	4.3	0	5	0	20

*p* - test probability value calculated by the Mann-Whitney test; NDI -Neck Disability Index

### Comparison of ODI and NDI by gender of students

The ODI and NDI indices are characterised by extreme asymmetry of distribution, with the median in some groups being 0, meaning that at least half of the respondents reported no complaints of back pain. The analysis of such a magnitude is not easy and obviously requires the use of non-parametric tests, and the description of the data should be based on the median and not on the mean (Tables 5 and 6).

Despite the rather small dispersion of the ODI and NDI values, significant differences between Poland and Portugal can be observed:

- for the ODI indicator among women - it is higher in the Polish group;
- for the NDI index among men - also higher in the Polish group.

**Table 5:** Comparison of oswestry disability index by sex of students

Country	ODI									
	Woman (*p* = 0.0239*)					Man (*p* = 0.2556)				
	Mean	Median	Std. dev.	***c***_25_	***c***_75_	Mean	Median	Std. dev.	***c***_25_	***c***_75_
**Poland**	3.2	1	4.5	0	6	2.5	0	4.3	0	3
**Portugal**	2.4	0	4.5	0	3	3.8	0	5.4	0	10

*p* - test probability value calculated by the Mann-Whitney test, *p* < 0.05 (*); ODI - Oswestry Disability Index

**Table 6:** Neck disability index comparison by sex of students

Country	NDI									
	Woman (*p* = 0.4428)					Man (*p* = 0.0470*)				
	Mean	Median	Std. dev.	***c***_25_	***c***_75_	Mean	Median	Std. dev.	***c***_25_	***c***_75_
**Poland**	3.3	2	4.5	0	5	3.5	2	4.4	0	5.5
**Portugal**	3.2	0.5	4.4	0	5	2.5	0	4.0	0	3

*p* - test probability value calculated by the Mann-Whitney test, *p* < 0.05 (*); NDI - Neck Disability Index

### COVID-19 incidence and pain severity

Below (Table 7) are the results of an analysis that tested whether the explanation of the existence of COVID-19 is associated with the occurrence of pain. Of course, the presence of such a correlation is not necessarily the evidence of a causal relationship. However, as it turns out, no correlation can be found between the declarative COVID-19 passage and complaints of back pain. No statistically significant correlations are found in the summary below.

**Table 7:** COVID-19 disease and back pain complaints

Sex	COVID-19 disease vs. ODI and NDI			
	Poland		Portugal	
	ODI	NDI	ODI	NDI
Woman	0.08 (*p* = 0.3751)	0.00 (*p* = 0.9908)	-0.1 (*p* = 0.2707)	-0.14 (*p* = 0.1372)
Man	0.05 (*p* = 0.5983)	0.09 (*p* = 0.3733)	-0.04 (*p* = 0.7734)	0.07 (*p* = 0.6201)

ODI - Oswestry Disability Index; NDI - Neck Disability Index

### Incidence of COVID-19 physical activity

There were no statistically significant associations between the number of COVID-19 cases and physical activity (Table 8). However, there was one statistically significant correlation (for males between walking and number of cases) and two near statistically significant correlations (for females, between moderate and full physical activity and the number of cases), but it is difficult to explain this in a logical way. Overall, the correlations are weak enough to stick with the conclusion that there is no association between the number of diseases and physical activity.

**Table 8:** COVID-19 disease and physical activity

IPAQ activity measures	COVID-19 disease and physical activity	
	Poland	Portugal
**Woman**		
Intense effort	0.14 (*p* = 0.119)	0.02 (*p* = 0.8028)
Moderate effort	0.15 (*p* = 0.087)	0.12 (*p* = 0.1814)
Walking	0.11 (*p* = 0.2034)	0.04 (*p* = 0.6688)
Total effort	0.17 (*p* = 0.0553)	0.06 (*p* = 0.5094)
**Man**		
Intense effort	0.15 (*p* = 0.138)	-0.1 (*p* = 0.4821)
Moderate effort	0.04 (*p* = 0.7257)	-0.07 (*p* = 0.6306)
Walking	0.25 (*p* = 0.0107*)	-0.23 (*p* = 0.1109)
Total effort	0.11 (*p* = 0.2926)	-0.18 (*p* = 0.2175)

*p* < 0.05 (*), IPAQ - International Physical Activity Questionnaire

### Back pain and physical activity

Some statistically significant correlations were found between physical activity and back complaints (Table 9). In the Polish female population (for Portugal only intensive physical activity) there is a tendency to report some complaints of the lumbar spine. Similar correlations are found in males, especially they are relatively strong and statistically significant, in the Portuguese group.

**Table 9:** Back pain and physical activity

IPAQ	Poland		Portugal	
	ODI	NDI	ODI	NDI
Woman				
Intense effort	0.31 (*p* = 0.0004***)	-0.11 (*p* = 0.2263)	0.24 (*p* = 0.01**)	0.08 (*p* = 0.4172)
Moderate effort	0.27 (*p* = 0.002**)	-0.05 (*p* = 0.6079)	0.05 (*p* = 0.5737)	-0.03 (*p* = 0.7109)
Walking	0.19 (*p* = 0.0308*)	-0.05 (*p* = 0.5944)	0.03 (*p* = 0.7109)	-0.06 (*p* = 0.5063)
Total effort	0.33 (*p* = 0.0001***)	-0.04 (*p* = 0.6766)	0.12 (*p* = 0.1788)	0.01 (*p* = 0.9548)
Man				
Intense effort	0.22 (*p* = 0.0297*)	0.31 (*p* = 0.0019**)	0.36 (*p* = 0.0106*)	-0.26 (*p* = 0.0687)
Moderate effort	0.09 (*p* = 0.3837)	0.17 (*p* = 0.0898)	0.31 (*p* = 0.0246*)	-0.3 (*p* = 0.0304*)
Walking	-0.06 (*p* = 0.5851)	0.04 (*p* = 0.6813)	0.26 (*p* = 0.0685)	-0.26 (*p* = 0.0681)
Total effort	0.14 (*p* = 0.1653)	0.26 (*p* = 0.0087**)	0.36 (*p* = 0.0105*)	-0.26 (*p* = 0.0701)

*p* < 0.05 (*); *p* < 0.01 (**); *p* < 0.001 (***); IPAQ - International Physical Activity Questionnaire; ODI - Oswestry Disability Index; NDI - Neck Disability Index

## Discussion

During the COVID-19 pandemic students were forced to reduce their physical activity and switch to a sedentary lifestyle, which was related to the introduction of online learning [6, 7, 20]. Studies conducted during the lockdown showed an increase in the incidence of back pain [5, 21]. A lower level of physical activity was one of the contributing factors [21, 22].

There are also some reports in the literature denying that COVID-19 lockdown [23] or introduced online learning [24] have a negative impact on spinal symptoms. At the same time, patients with COVID-19 infection have been shown to have increased neck and back pain, which may persist after infection [25].

Researchers investigated the extent of back pain and physical activity in students in two parts of Europe, Poland and Portugal, in the year following the announcement of the COVID-19 pandemic. It was observed that there were no statistically significant differences in pain levels between respondents from the two countries. With the exception of the NDI for Portugal, at least half of the students reported no pain complaints. Despite the rather small divergence in ODI and NDI values, significant differences are observed between Poland and Portugal (the ODI index in women is higher in the Polish group, and the NDI index in men is also higher in the Polish group). This is in line with global trends. In the studies from the pandemic period, there is a trend toward relatively equal prevalence of cervical and lumbar pain [21, 26]. Among these, lumbar spine pain predominates in women [27]. Gender differences in the prevalence of sacral pain may be related to physiological factors [28] as well as sociological factors [29].

However, as it turned out, no association was found between the declarative passage of COVID-19 and complaints of low back pain in the study group. There was also no statistically significant association between the number of COVID-19 cases and physical activity.

Physical inactivity is directly associated with an increased risk of back pain [30–32]. In our study, statistically significant correlations were found between physical activity and back pain. In the Polish female population (for Portugal, only intense physical activity), there is a tendency to declare some complaints of the lumbosacral spine. It is difficult to interpret this relationship unambiguously. Similar correlations are found in men, and in the Portuguese group in particular they are relatively strong and statistically significant. However, in the Portuguese men, negative correlations between activity and NDI levels are also shown, and these correlations are easier to justify from a content point of view - because physical activity should have a positive effect on the function of the cervical spine segment, which is particularly vulnerable to pain caused, for example, by sitting in front of a monitor or a TV. Studies found in the literature also confirm that more severe cervical pain is positively correlated with less physical activity, which is confirmed by the results of studies conducted among university students before [33] and also during [22] the pandemic. First, because of the cross-sectional nature of the study, no causal inferences can be made about the observed associations. A second limitation was the small sample group and the conduct of the study in an online format.

In our study, there were no statistically significant associations between the number of COVID-19 cases and physical activity. However, there was one statistically significant correlation and two near-significant correlations, but it is difficult to explain this in a logical way. Overall, the correlations are weak enough to support the conclusion that there is no association between the number of diseases and physical activity. In a systematic review and meta-analysis on changes in physical activity patterns Due to the COVID-19 pandemic, we found that most self-reported and all device-based measurement methods showed a reduction in physical activity levels. However, the effects were not found to be significant in all age groups, as in our own study [34]. Another rapid review on the impact of COVID-19 on physical activity also noted a few studies that reported contradicting results [35].

The present study has some limitations. Firstly, the cross-sectional nature of the study does not allow causal inferences to be drawn on the links detected. A second limitation was the small sample group (there may be potential selection bias in the study due to examining a less representative sample of the population, which may have led to inaccurate results) and the conduct of the study in an online format. Despite the limitations, our study also has strengths, namely the standardised instruments used and the ease of access to the study group.

## Conclusions

In the year after the announcement of the pandemic COVID-19 there is still a significant prevalence of back pain among students in Poland and Portugal. This study complements the literature on spinal pain and physical activity during the pandemic COVID-19. Interpretation of the results obtained from the pilot study may be clinically useful, because provides a rationale for the appropriateness of further monitoring of physical activity status and spinal complaints among students in Poland and Portugal. In addition, the authors believe that it is necessary to implement not only effective strategies to prevent the onset of back pain but also broad-based prevention in students. One of the elements of this prevention is maintaining an appropriate level of physical activity.

## Data Availability

The data that support the findings of this study are openly available in RepOD at 10.18150/0J3FKR.

## Abbreviations

COVID-19: coronavirus disease 2019
IPAQ: International Physical Activity Questionnaire
NDI: Neck Disability Index
ODI: Oswestry Disability Index
